# Combination bromo- and extraterminal domain and poly (ADP-ribose) polymerase inhibition synergistically enhances DNA damage and inhibits neuroblastoma tumorigenesis

**DOI:** 10.1007/s12672-022-00563-5

**Published:** 2022-10-13

**Authors:** Jillian C. Jacobson, Jingbo Qiao, Rachael A. Clark, Dai H. Chung

**Affiliations:** Department of Pediatric Surgery, University of Texas Southwestern Medical Center and Children’s Health, 1935 Medical District Dr. Mailstop F3.66, Dallas, TX 75235 USA

**Keywords:** Neuroblastoma, BET, PARP, JQ1, Olaparib

## Abstract

**Purpose:**

JQ1 is a bromo- and extraterminal (BET) domain inhibitor that downregulates *MYC* expression and impairs the DNA damage response. Poly (ADP-ribose) polymerase (PARP) inhibitors prevent DNA damage sensing and repair. We hypothesized that JQ1 would promote a DNA repair-deficient phenotype that sensitizes neuroblastoma cells to PARP inhibition.

**Methods:**

Four human neuroblastoma cell lines were examined: two *MYCN*-amplified (BE(2)-C and IMR-32), and two non-*MYCN*-amplified (SK-N-SH and SH-SY5Y). Cells were treated with JQ1 (BET inhibitor), Olaparib (PARP inhibitor), or in combination to assess for therapeutic synergy of JQ1 and Olaparib. Treated cells were harvested and analyzed. Quantitative assessment of combination treatment synergy was performed using the median effect principle of Chou and Talalay.

**Results:**

Combination treatment with Olaparib decreased the IC_50_ of JQ1 by 19.9-fold, 2.0-fold, 12.1-fold, and 2.0-fold in the BE(2)-C, IMR-32, SK-N-SH, and SH-SY5Y cell lines, respectively. In the *MYCN-*amplified cell lines, BE(2)-C and IMR-32, combination treatment decreased gene expression of *MYCN* relative to single-drug treatment alone or control. Combination treatment decreased protein expression of DNA repair proteins Ku80 and RAD51, led to accumulation of DNA damage marker phospho-histone H2A.X, and increased caspase activity. In the non-*MYCN*-amplified cell lines, SK-N-SH and SH-SY5Y, combination treatment induced G0/G1 cell cycle arrest.

**Conclusions:**

Combination BET and PARP inhibition synergistically inhibited neuroblastoma tumorigenesis in vitro. In *MYCN*-amplified neuroblastoma cells, this effect may be induced by downregulation of *MYCN* transcription, defects in DNA repair, accumulation of DNA damage, and apoptosis. In non-*MYCN*-amplified cell lines, combination treatment induced cell cycle arrest.

**Supplementary Information:**

The online version contains supplementary material available at 10.1007/s12672-022-00563-5.

## Introduction

Despite advances in treatment, high-risk neuroblastoma (NB) accounts for approximately 15% of cancer-related mortality in children with a five-year survival rate of less than 40% [1]. Furthermore, despite more aggressive therapeutic regimens, which are associated with increased treatment morbidity, the overall prognosis for high-risk NB remains dismal with high rates of recurrence and many tumors demonstrating resistance to current therapies, including chemotherapy [2]. Therefore, there is a substantial need for novel therapeutic options.

*MYCN* amplification, a feature of high-risk NB due to its role in promoting NB tumorigenesis and proliferation, has made transcription factors of oncoproteins such as N-myc attractive potential therapeutic targets. However, these oncoproteins have historically been resistant to conventional treatment strategies due to a paucity of high-affinity binding sites [3]. Moreover, designing novel small molecules to specifically disrupt or recruit protein–protein or protein-DNA interactions has been a challenging requirement [4]. This has led to further study and development of epigenetic modifiers such as the bromo- and extraterminal (BET) domain family of proteins, composed of bromodomain-containing protein 2 (BRD2), BRD3, BRD4, and bromodomain testis-specific protein (BRDT) [5, 6]. BET proteins are regulators of transcription that play vital roles in homeostasis, cell cycle progression, DNA repair (particularly repair of double strand DNA breaks), and cell survival [7–9]. BET inhibitors have demonstrated efficacy in treating both solid tumor and hematologic malignancies in preclinical models [7]. In preclinical models of NB, BET inhibitors such as JQ1, a novel thienotriazolo-1,4-diazepine, have been demonstrated to downregulate *MYC* expression by displacing BET bromodomains from chromatin after selectively recognizing and competitively binding histone acetylated lysine residues [5, 6]. JQ1 has demonstrated efficacy in inhibiting N-myc expression and cell proliferation, as well as inducing differentiation and apoptosis in preclinical models of NB [5, 6, 10, 11]. Despite these results, clinical testing and application of BET inhibitors for various cancers has been limited by unexpected dose-limiting toxicity [7, 12].

Poly(ADP-ribose) polymerase (PARP) proteins play a vital role in numerous cell processes including DNA repair, cell signaling, and cell death [13]. PARP inhibitors, including Olaparib, have been approved by the United States Food and Drug Administration (FDA) as anti-cancer therapy for numerous malignancies, including breast, ovarian, prostate, and pancreatic cancer [14]. PARP proteins, especially PARP1, function as DNA damage sensors, binding to sites of DNA strand breaks, PARylating histones, and inducing the recruitment of effectors of DNA repair, only unbinding once DNA is repaired [13, 15–17]. In tumor cells, this process of DNA damage repair can also prevent cell death [17, 18]. PARP inhibitors prevent PARylation and trap PARP onto DNA [19]. This can enhance DNA damage and also convert single-strand lesions on DNA to double-strand breaks, therefore enhancing dependence on double-strand break repair pathways such as homologous recombination (HR) and non-homologous end-joining (NHEJ) [13, 15, 20, 21]. Recently, increased PARP1 and PARP2 expression have been found to be significantly associated with high-risk NB and predictive of poor survival [16]. Furthermore, PARP inhibition was found to enhance replication stress, leading to the accumulation of DNA damage, and ultimately inducing mitotic catastrophe in *MYCN-*amplified NB cells in vitro [16]. Tumors that are deficient in HR are extremely sensitive to PARP inhibitors [13]. This has prompted efforts to identify drug combinations that impair HR or create DNA repair-deficient phenotypes in cancer cells in order to enhance tumor sensitivity to PARP inhibitors [13].

A combination of BET and PARP inhibition has demonstrated efficacy in inhibiting tumorigenesis in preclinical models of multiple cancer types, including pancreatic, breast, ovarian, prostate, and lung cancers [13, 15, 17, 22]. However, current studies have largely been limited to cancers with known defects in HR. Despite preclinical studies demonstrating the efficacy of BET and PARP inhibitors individually against NB tumorigenesis, to our knowledge, this is the first study evaluating whether BET and PARP inhibition could function synergistically in combination against NB tumorigenesis. We hypothesized that the BET inhibitor JQ1 would promote a DNA repair-deficient phenotype, sensitizing NB cells to PARP inhibition by Olaparib, and sought to determine how *MYCN* amplification status may affect the efficacy of this combination therapy.

## Materials and methods

### Antibodies and reagents

Primary antibodies against Neurofilament-M (NF-M)(#2838), caspase3 (#9662, #9664), pAKT (#4060), AKT (#4685), p53 (#2524), N-myc (#94055), C-myc (#5605), Ku80 (#2180), RAD51 (#8875), phospho-histone H2A.X (Ser139) (#2577), and PTEN (#9118) were purchased from Cell Signaling Technology (Danvers, MA, USA). Primary antibody against neuron-specific enolase (NSE) (ab53025) was obtained from Abcam (Cambridge, UK). Primary antibody against p21 (#610234) was obtained from BD Bioscience (Franklin Lakes, NJ, USA). Primary antibodies against β-actin and Ponceau S solution were obtained from Sigma-Aldrich (St. Louis, MO). Anti-GAPDH antibody (#20357) and secondary anti-mouse, anti-rabbit, and anti-goat antibodies were obtained from Santa Cruz Biotechnology, Inc (Santa Cruz, CA, USA).

### Cell lines and culture

The human NB cell lines SK-N-SH, SH-SY5Y, BE(2)-C and IMR-32 were purchased from the American Type Culture Collection (Manassas, VA). Cells were maintained in Rockwell Park Memorial Institute (RPMI) culture medium 1640 with 10% fetal bovine serum (FBS) at 37 °C in a humidified atmosphere consisting of 5% CO_2_ and 95% air.

### Cell viability assay

SK-N-SH, SH-SY5Y, BE(2)-C, and IMR-32 cells were plated in 96-well plates at 3 × 10^3^, 2 × 10^3^, 1 × 10^3^, and 3 × 10^3^ cells per well, respectively, in RPMI culture medium with 10% FBS and allowed to attach overnight. Cells were subsequently treated with JQ1, Olaparib, or both. Cell viability with combination treatment was measured after same day treatment with both drugs for all four cell lines. The SK-N-SH, BE(2)-C, and IMR-32 cell lines also underwent a second experiment evaluating the effects of staggered treatment (JQ1 followed by Olaparib 24 h later). Cell viability was measured using Cell Counting Kit-8 (CCK-8) colorimetric assay (Dojindo Molecular Technologies, Inc., Rockville, MD, USA) 72 h after last treatment dose for each treatment group.

### Microscopy

Each cell line was plated at two different cell densities in 6-well plates. SK-N-SH, SH-SY5Y, BE(2)-C, and IMR-32 cells were plated at 8000 and 16,000, 3000 and 10,000, 5000 and 10,000, and 100,000 and 150,000 cells per well, respectively. Cells were allowed to attach for 48 h prior to treatment with JQ1, Olaparib, or combination. JQ1 and Olaparib dosages were 300 nM for the SK-N-SH and BE(2)-C cell lines and 150 nM for the IMR-32 cell line. The SH-SY5Y cell line was treated with 200 nM of JQ1 and 500 nM of Olaparib. Cells were observed for five to seven days after treatment to evaluate for changes in treatment-induced morphology. Images were captured using a BioTek Cytation 5 Cell Imaging Multi-Mode Reader (Agilent, Santa Clara, CA, USA).

### Clonogenic assay

SK-N-SH, BE(2)-C, and IMR-32 cells were plated at 3000, 1000, and 10,000 cells per well, respectively, on 12-well plates in triplicate. 48 h after plating, cells were treated with JQ1, Olaparib, or both. The JQ1 dose was 50 nM for the SK-N-SH and BE(2)-C cell lines and 125 nM for the IMR-32 cell line. The Olaparib dose was 100 nM for the SK-N-SH and IMR-32 cell lines and 1000 nM for the BE(2)-C cell line. Cells were allowed to grow for 6–10 days after treatment. SK-N-SH and BE(2)-C colonies were stained with 0.01–0.05% crystal violet dye, photographed, and counted using the Bio-Rad Gel Doc XR + Imager (Bio-Rad, Hercules, CA, USA).

Due to the IMR-32 cell line’s propensity to detach from wells with washing, cells were alternatively trypsinized, centrifuged, resuspended in fresh media, and placed on dual chamber slides with trypan blue dye for cell counting using the Bio-Rad TC10 Automated Cell Counter (Bio-Rad, Hercules, CA, USA).

In order to assess for a dose-dependent response to treatment using clonogenic assays, the SK-N-SH and BE(2)-C cell lines were also plated at 1500 and 500 cells per well, respectively, in 24-well plates in triplicate. Cells were treated with varying doses of JQ1, Olaparib, or combination treatment 48 h after plating. SK-N-SH and BE(2)-C cells were allowed to grow for 8 or 5 days after treatment, respectively, prior to staining with 0.02% crystal violet dye, photographing, and colony counting.

### RNA isolation and qPCR with reverse transcription

Total RNA was isolated and purified using a Trizol Reagent (Thermo Scientific, Waltham, MA). The High-Capacity cDNA Reverse Transcription Kit (Applied Biosystems, Carlsbad, CA) was used to synthesize complementary DNA. Reverse transcription quantitative polymerase chain reaction (RT-qPCR) was performed using iTaq Universal SYBR Green Supermix (Bio-Rad, Hercules, CA), and data were collected with a CFX96 instrument (Bio-Rad, Hercules, CA, USA). Results were normalized to an endogenous control, *ACTB*. Amplification was performed for 40 cycles of 30 s at 95 °C, 30 s at 55 °C, and 40 s at 72 °C. Primers used to detect the expression of qPCR were the following: *MYCN* (forward: 5’-GCTTCTTACCCGGACGAAGATG-3’; reverse: 5’-CAGCTCGTTCTCAAGCAGCAT), *PTEN* forward: 5’-CGGGCTCAGGCGAGGGAGAT-3’, reverse: 5’-GCCCACGGCTCCACCTTCC-3’), *ACTB* (forward: 5’-GAGCGCGGCTACAGCTT-3’; reverse: 5’-TCCTTAATGTCACGCACGATTT-3’).

### Immunoblotting

Cells were collected using cell lysis buffer and denatured samples were prepared for immunoblotting, as we have previously described [23, 24]. Equal amounts of protein were loaded and separated by NuPAGE 4–12% Bis–Tris gel, followed by transfer onto PVDF membranes (Bio-Rad, Hercules, CA, USA). Membranes were blocked with 5% nonfat milk in TBS-T for one hour at room temperature. The blots were then incubated with antibodies against the human target proteins by using rabbit or mouse anti-human antibodies (1:500 – 2000 dilution) overnight at 4 °C. Anti-rabbit or anti-mouse secondary antibodies conjugated with HRP were incubated for one hour and visualized using an enhanced chemiluminescence detection system (PerkinElmer, Waltham, MA, USA). Densitometry was used to assess quantitative protein expression using ImageJ software (Rasband, W.S., ImageJ, U.S. National Institutes of Health, Bethesda, Maryland, USA, https://imagej.nih.gov/ij/, 1997–2018).

### Cell cycle analysis and flow cytometry

SK-N-SH and SH-SY5Y cells were plated separately in 6-well plates at 180,000 cells per well and allowed to attach overnight. Cells in the treatment group received a combination of JQ1 (200 nM) and Olaparib (500 nM) at 0 and 24 h time points. Samples were collected 16, 24, and 48 h after initial treatment. Samples were trypsinized, centrifuged, washed and resuspended in phosphate-buffered saline (PBS), and fixed in 70% ethanol at 4 °C. Prior to cell staining, samples were centrifuged and resuspended in PBS. RNase, DNase-free was added to each sample and subsequently incubated at 37 °C for 30 min. Samples were chilled on ice prior to staining with propidium iodide. Stained cells were analyzed with the flow cytometer (BD Accuri C6 + , BD Biosciences, Franklin Lakes, NJ).

### Immunohistochemistry and immunofluorescence staining

The phospho-histone H2A.X (Ser139) antibody was obtained from Cell Signaling Technology (Danvers, MA, USA). BE(2)-C cells were plated on coverslips in a 12-well plate at a density of 2–6 × 10^3^ cells per well and allowed to attach overnight. Cells were treated as part of one of four treatment groups (control, JQ1 (1000 nM), Olaparib (3000 nM), or combination) and cultured for 72 h. Cells were subsequently washed with PBS and fixed with 4% formaldehyde in 1× PBS at room temperature. They were then washed in 1 × PBS three times prior to being blocked in blocking buffer for one hour. Primary antibody was subsequently applied at a concentration of 1:400 and incubated at room temperature for 1.5 h. The samples were again washed three times in 1× PBS prior to incubation with Alexa Fluor 594 dye-tagged secondary antibody (Life Technologies, Grand Island, NY) at a concentration of 1:500 for 30 min. Samples were again washed in 1× PBS three times. Coverslips were mounted and slides were left to dry. 4’,6-diamidino-2-phenylindole (DAPI) was used for staining nuclei. Images were captured using a Biotek Cytation 5 Cell Imaging Multi-Mode Reader (Agilent, Santa Clara, CA, USA).

### Quantification of therapeutic synergy

Therapeutic synergy of combination BET and PARP inhibition was quantitatively assessed using the Chou-Talalay calculation and Compusyn software (ComboSyn, Inc., Paramus, NJ, USA) [25].

### Statistical analysis and experimental analysis

All experiments were repeated in triplicate. The scoring index and relative expression values were expressed as mean ± SEM or SD. Statistical analyses were performed using Student’s and Welch’s *t*-tests, Mann–Whitney U tests, and analyses of variance using GraphPad Prism (version 9.4.1, GraphPad Software, San Diego, California USA, www.graphpad.com). A *p* value ≤ 0.05 was considered significant.

## Results

### Combination treatment with JQ1 and Olaparib synergistically inhibited NB cellular proliferation

**Fig. 1:**
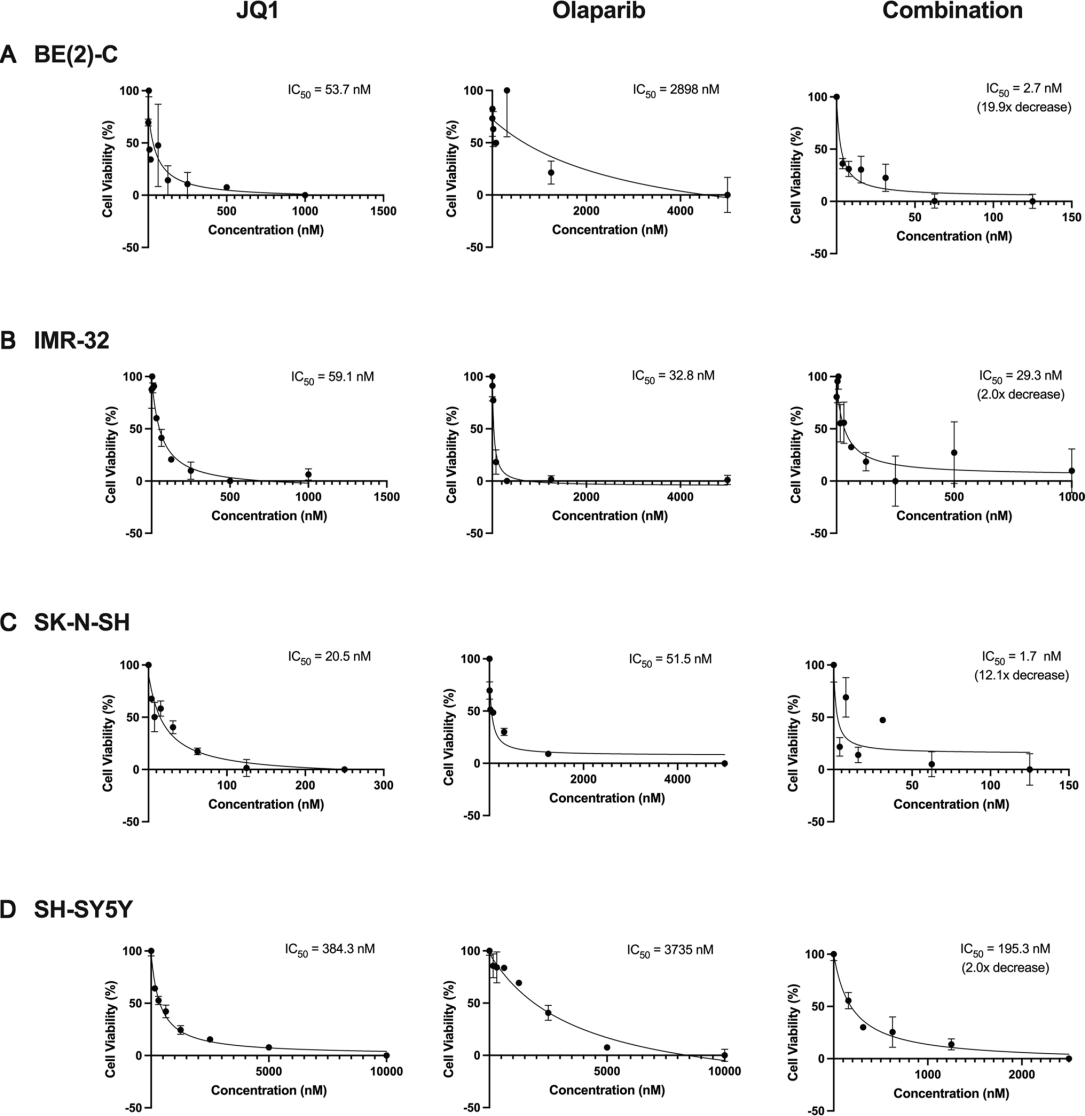
Combination treatment with BET inhibitor JQ1 and PARP inhibitor Olaparib inhibited NB cellular proliferation in vitro and decreased the IC_50_ of JQ1. Cell viability was measured 72 h after same-day combination treatment using CCK-8 assays. **A** Combination treatment of BE(2)-C neuroblastoma cells with JQ1 and Olaparib (2898 nM) decreased the IC_50_ dose of JQ1 19.9-fold. **B** Combination treatment of IMR-32 neuroblastoma cells with JQ1 and Olaparib (32.8 nM) decreased the IC_50_ dose of JQ1 2.0-fold. **C** Combination treatment of SK-N-SH neuroblastoma cells with JQ1 and Olaparib (51.5 nM) decreased the IC_50_ dose of JQ1 12.1-fold. **D** Combination treatment of SH-SY5Y neuroblastoma cells with JQ1 and Olaparib (3735 nM) decreased the IC_50_ dose of JQ1 2.0-fold

We first examined whether BET inhibition with JQ1 plus PARP inhibition with Olaparib efficaciously inhibits NB cellular proliferation in vitro using CCK-8 assays 72 h after treatment. Two *MYCN*-amplifying NB cell lines, BE(2)-C and IMR-32, and two non-*MYCN*-amplifying NB cell lines, SK-N-SH and SH-SY5Y, were treated with JQ1 alone and Olaparib alone to identify the IC_50_ doses of each drug. Each cell line was subsequently treated using increasing doses of JQ1 in combination with each cell line’s respective IC_50_ dose of Olaparib. When treated on the same day in combination, the IC_50_ dose of JQ1 decreased 19.9-fold, 2.0-fold, 12.1-fold, and 2.0-fold in the BE(2)-C (Fig. 1A), IMR-32 (Fig. 1B), SK-N-SH (Fig. 1C), and SH-SY5Y (Fig. 1D) cell lines, respectively.

We subsequently tested whether treating each cell line with JQ1 24 h prior to treatment with Olaparib could sensitize NB cells and enhance this inhibitory effect in the BE(2)-C, IMR-32, and SK-N-SH cell lines. When staggering treatment with these two drugs by 24 h, the IC_50_ of JQ1 increased 6.4-fold in the BE(2)-C cell line and decreased 1.7-fold and 51.3-fold in the IMR-32 and SK-N-SH cell lines, respectively (Additional file 1: Fig. S1). The increase in IC_50_ seen in the BE(2)-C cell line may reflect the relatively more rapid doubling time of this cell line. Together, this data suggest that the optimal timing of combination treatment may vary by cell line.

### Treatment with JQ1 and Olaparib induces differentiation, as demonstrated by neurite outgrowth

Previous studies have shown that treatment with JQ1 induces neuroblastoma cell differentiation in BE(2)-C cells, demonstrated by morphologic changes including a polarized phenotype with longer neurites, as well as increased protein expression of neural differentiation markers ZNF423 and NF-M [5].

**Fig. 2:**
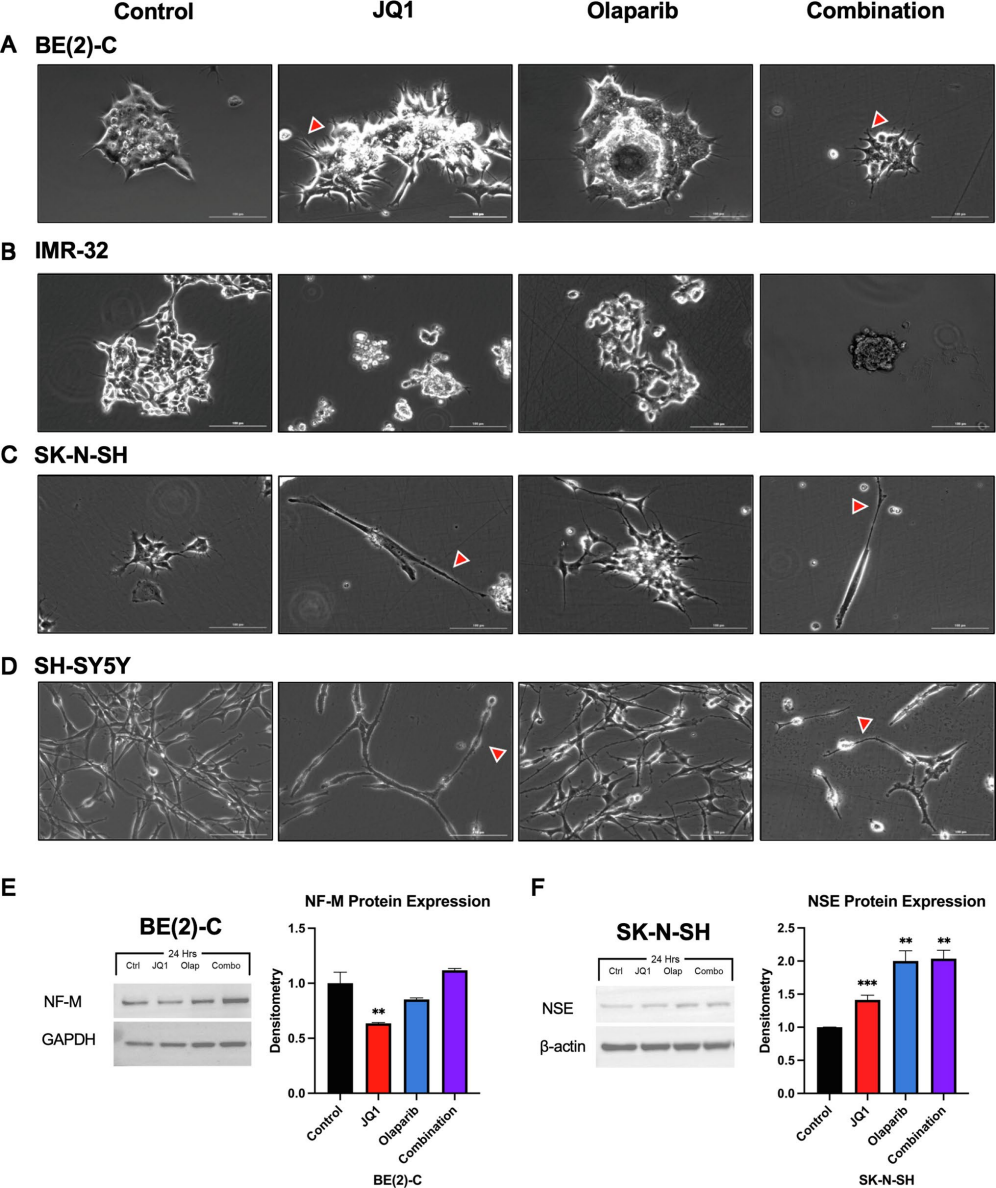
Combination treatment with BET inhibitor JQ1 and PARP inhibitor Olaparib induced changes in cellular morphology, including differentiation, as demonstrated by neurite outgrowth. **A**–**D** Human neuroblastoma BE(2)-C (**A**), IMR-32 (**B**), SK-N-SH (**C**), and SH-SY5Y **D** cells were plated as monolayers in serum-supplemented media and treated for five to seven days. All cell lines demonstrated changes in morphology with drug treatment. Both *MYCN*-amplifying and non-*MYCN*-amplifying cell lines demonstrated changes consistent with neural differentiation, including the extension of neurites *(red arrowheads)*, after treatment with JQ1, as well as combination treatment with JQ1 and Olaparib. Scale bar: 100 μm. **E** Combination treatment induced a 1.12-fold increase in NF-M protein expression in BE(2)-C cells after 24 h of treatment, as demonstrated by immunoblotting. **F** Combination treatment induced a 2.0-fold increase in NSE protein expression in SK-N-SH cells after 24 h of treatment, as demonstrated by immunoblotting (*p* = .004). (Statistical analysis was performed using Welch’s t-tests to evaluate for differences in means between treatment groups. Mean ± SEM; * = *p* ≤ .05 vs. control, ** = *p* ≤ .01 vs. control, *** = *p* ≤ .001 vs. control)

Using microscopy, we found that both *MYCN*-amplifying and non-*MYCN*-amplifying cells demonstrated changes in morphology after treatment with JQ1, as well as after combination treatment with JQ1 and Olaparib (Fig. 2A–D). Specifically, they demonstrated changes consistent with neural differentiation, including the extension of neurites.

This was also associated with a 1.12-fold increase in neurofilament-medium (NF-M) protein expression in the BE(2)-C cell line and a 2.0-fold increase in neuron-specific enolase (NSE) protein expression in the SK-N-SH cell line with combination treatment at 24 h, demonstrated by immunoblotting with densitometry in Fig. 2E, F respectively.

### Combination treatment with JQ1 and Olaparib synergistically inhibited colony formation in vitro

**Fig. 3:**
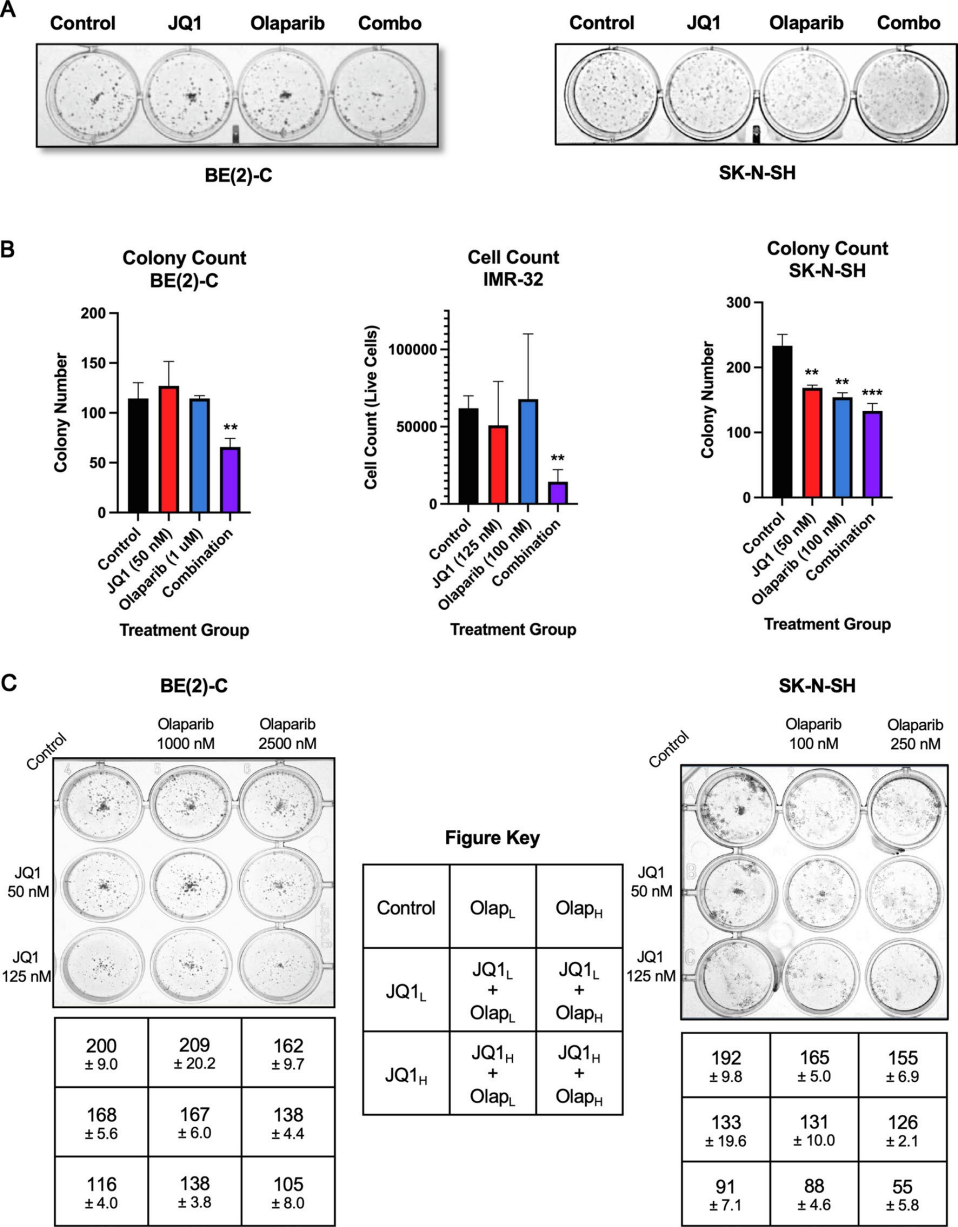
Combination treatment with BET inhibitor JQ1 and PARP inhibitor Olaparib synergistically inhibited colony formation in vitro. **A** Clonogenic assays were performed on BE(2)-C and SK-N-SH cells separated into four treatment groups: control, JQ1, Olaparib, or combination treatment. The treatment dose of JQ1 was 50 nM for both cell lines. The treatment dose of Olaparib was 1 μM for BE(2)-C cells and 100 nM for SK-N-SH cells. Figures are representative images of colonies formed from single cell proliferation. **B** Clonogenic assay experiments represented in Figure A were assessed and quantified for the BE(2)-C and SK-N-SH cell lines. Due to the IMR-32 cell line’s propensity to detach with washing, in lieu of washing and staining with crystal violet dye to obtain colony counts, cells were alternatively trypsinized, centrifuged, resuspended in fresh media, and placed on dual chamber slides with trypan blue dye for cell counts. The JQ1 and Olaparib doses for the IMR-32 cell line were 125 nM and 100 nM, respectively. Statistical significance was performed using independent samples *t*-tests to compare means of each treatment group relative to control. (Mean ± SEM; * = *p* ≤ .05 vs. control, ** = *p* ≤ .01 vs. control, *** = *p* ≤ .001 vs. control). **C** To assess for a potential dose-dependent response to single and combination drug therapy, BE(2)-C and SK-N-SH cells were plated in 24-well plates. Each cell line was treated with a low dose of each drug (i.e., JQ1_L_, Olap_L_), as well as a dose 2.5 times higher in concentration (i.e., JQ1_H_, Olap_H_). Each cell line was also treated with varying combinations of these doses (i.e., JQ1_L_ + Olap_L_, JQ1_H_ + Olap_L_, JQ1_L_ + Olap_H_, or JQ1_H_ + Olap_H_). As expected, each cell line demonstrated a dose-dependent response to single drug and combination treatment, with fewer colonies *(mean* ± *SD)* formed in the high-dose single drug treatment relative to low-dose, and JQ1_H_ + Olap_H_ treatment group demonstrating the lowest number of colonies overall for each cell line. In the BE(2)-C cell line, the JQ1_L_ + Olap_L_ and JQ1_H_ + Olap_H_ treatment groups demonstrated a 16.5% and 47.5% decrease in colony formation relative to control. In the SK-N-SH cell line, the JQ1_L_ + Olap_L_ and JQ1_H_ + Olap_H_ treatment groups demonstrated a 31.8% and 71.4% decrease in colony formation relative to control. Figures are representative images of colonies formed from single cell proliferation

Clonogenic assays were performed on the BE(2)-C and SK-N-SH cell lines to quantitatively assess colony formation in cells treated with JQ1 alone, Olaparib alone, or both drugs in combination relative to control. Combination treatment significantly decreased colony formation in both the BE(2)-C and SK-N-SH cell lines with better efficacy than either drug treatment alone. Figure 3A demonstrates colony formation after staining with 0.01–0.05% crystal violet dye. Combination treatment resulted in a 42.5% and 43.1% decrease in colony formation in the BE(2)-C and SK-N-SH cell lines, respectively, relative to control (*p* = 0.0097 and *p* = 0.0012, respectively)(Fig. 3B).

Due to the IMR-32 cell line’s propensity to detach from wells with washing, in lieu of staining as described above for colony counting, cells were typsinized, aspirated, centrifuged, resuspended in fresh media, and placed on dual chamber slides with trypan blue dye for cell counting using the Bio-Rad TC10 Automated Cell Counter (Bio-Rad, Hercules, CA, USA). Combination treatment of IMR-32 with JQ1 and Olaparib decreased the number of viable cells by 78.6% relative to control (*p* = 0.0019)(Fig. 3B).

To assess for a possible dose-dependent response to treatment, colony counts were also performed in the BE(2)-C and SK-N-SH cell lines after treatment with increasing doses of JQ1, Olaparib, or combination therapy (Fig. 3C). Each cell line was plated in 24-well plates and treated with a low dose of each drug (i.e., JQ1_L_, Olap_L_), as well as a dose 2.5 times higher in concentration (i.e., JQ1_H_, Olap_H_). Each cell line was then treated with varying combinations of these doses (i.e., JQ1_L_ + Olap_L_, JQ1_H_ + Olap_L_, JQ1_L_ + Olap_H_, or JQ1_H_ + Olap_H_). As expected, each cell line demonstrated a dose-dependent response to single drug and combination treatment. Fewer colonies were formed after high dose single drug treatment relative to low dose single drug treatment relative to low dose for each drug and the JQ1_H_ + Olap_H_ treatment group demonstrated the lowest number of colonies overall for each cell line. In the BE(2)-C cell line, the JQ1_L_ + Olap_L_ and JQ1_H_ + Olap_H_ treatment groups demonstrated a 16.5% and 47.5% decrease in colony formation relative to control. In the SK-N-SH cell line, the JQ1_L_ + Olap_L_ and JQ1_H_ + Olap_H_ treatment groups demonstrated a 31.8% and 71.4% decrease in colony formation relative to control.

### BET inhibition downregulated *MYCN* transcription and N-myc protein translation in *MYCN*-amplifying NB cell lines with the greatest effect observed with combination treatment with PARP inhibition

**Fig. 4:**
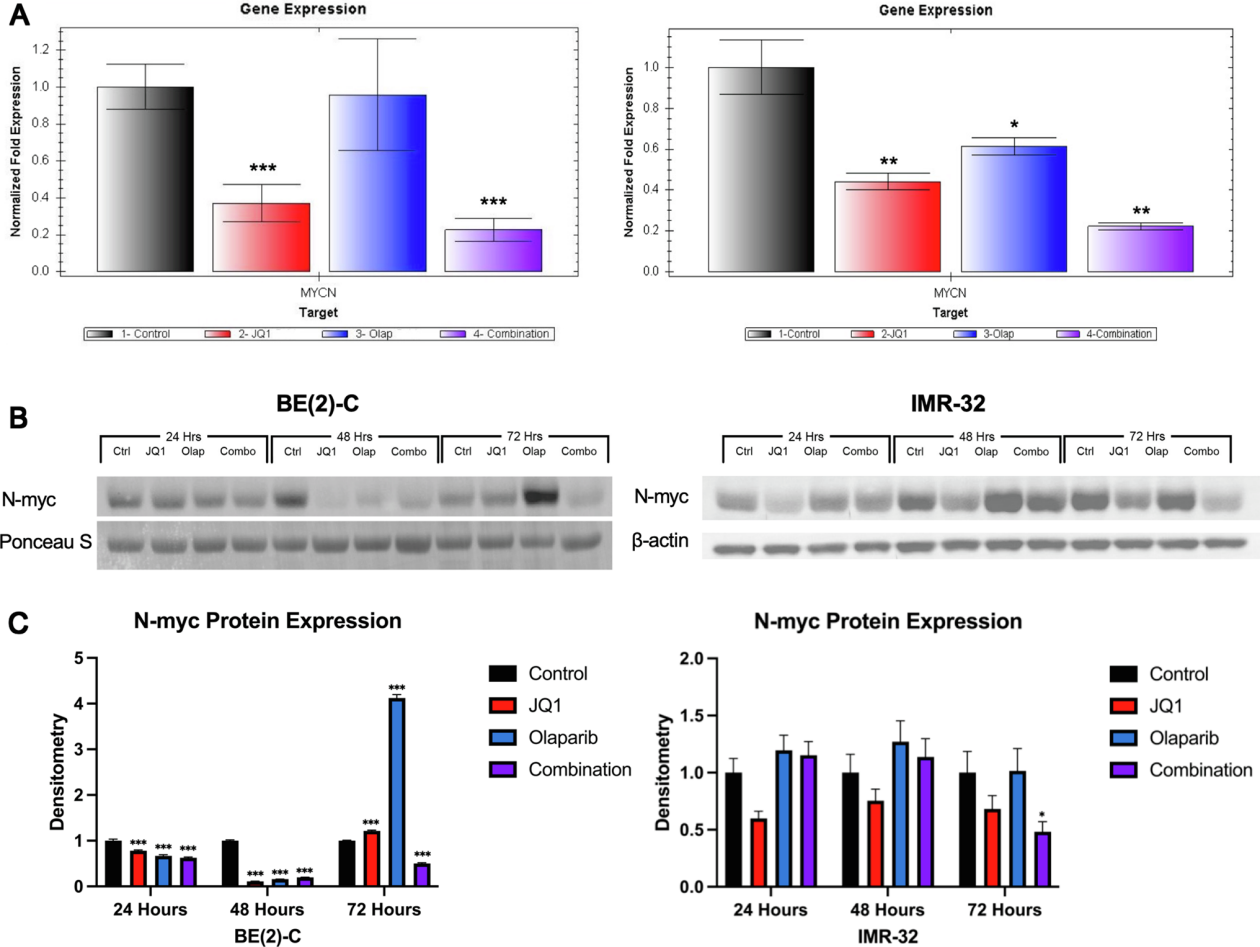
Combination treatment with BET inhibitor JQ1 and PARP inhibitor Olaparib downregulated *MYCN* transcription and N-myc protein expression. Although this effect appears to be induced by JQ1, greatest effect was seen with combination treatment. **A** Treatment with JQ1 alone was associated with a 2.70-fold and 2.25-fold decrease in *MYCN* gene expression relative to control in the BE(2)-C and IMR-32 cell lines, respectively (*p* = .0002 and *p* = .0129, respectively). Combination treatment was associated with a 4.48-fold and 4.51-fold decrease in *MYCN* gene expression relative to control in the BE(2)-C and IMR-32 cell lines, respectively (*p* = .000008 and *p* = .0028, respectively). (Mean ± SEM; * = *p* ≤ .05 vs. control, ** = *p* ≤ .01 vs. control, *** = *p* ≤ .001 vs. control). **B** Combination treatment decreased N-myc protein expression in BE(2)-C and IMR-32 cells, as seen by Western blotting. **C** Densitometry analysis of N-myc protein expression was performed and presented as a ratio of N-myc protein band density relative to the density of each housekeeping control band. BE(2)-C cells treated with combination therapy demonstrated a sustained decrease in N-myc protein expression at 37.5%, 80.3%, and 49.9% at 24, 48, and 72 h, respectively. IMR-32 cells treated with combination therapy demonstrated a 51.8% decrease in N-myc protein expression relative to control at 72 h. (Mean ± SEM; * = *p* ≤ .05 vs. control, ** = *p* ≤ .01 vs. control, *** = *p* ≤ .001 vs. control)

BET inhibition with JQ1 has been demonstrated to displace BRD4 from the *MYCN* promoter, leading to decreased transcription of *MYCN* [6, 26]. The effects of BET inhibition, PARP inhibition, and combination treatment on *MYCN* expression were therefore evaluated using RT-qPCR. We found that BE(2)-C and IMR-32 NB cells treated with JQ1 demonstrated decreased expression of *MYCN* with lowest expression seen in cells treated with both JQ1 and Olaparib (Fig. 4A). The BE(2)-C cell line demonstrated a 2.70-fold and 4.48-fold decrease in *MYCN* expression with JQ1 and combination treatment, respectively (*p* = 0.0002 and *p* = 0.000008, respectively). The IMR-32 cell line demonstrated a 2.25-fold and 4.51-fold decrease in *MYCN* expression with JQ1 and combination treatment, respectively (*p* = 0.0129 and *p* = 0.0028, respectively).

Immunoblotting was performed to assess the effects of treatment on N-myc protein expression at 24, 48, and 72 h (Fig. 4B). BE(2)-C cells treated with JQ1 demonstrated a 21.7% and 88.8% decrease in N-myc protein expression relative to control at 24 and 48 h, respectively, followed by a 21.4% increase at 72 h. BE(2)-C cells treated with combination therapy demonstrated a sustained decrease in N-myc protein expression at 37.5%, 80.3%, and 49.9% at 24, 48, and 72 h, respectively (Fig. 4C). Likewise, in the IMR-32 cell line, JQ1 induced a 40.1%, 24.56%, and 32.0% decrease in N-myc protein expression relative to control at 24, 48, and 72 h, respectively, likely contributing to the 51.8% decrease in N-myc protein expression seen in the combination treatment group at 72 h (Fig. 4C). The increase in N-myc protein expression seen with Olaparib treatment relative to control in the BE(2)-C cells at 72 h and at each time point in the IMR-32 cells suggests that the decrease in N-myc protein expression seen with combination treatment is driven by JQ1, specifically.

### Combination BET and PARP inhibition caused apoptosis in *MYCN*-amplifying NB cells

**Fig. 5:**
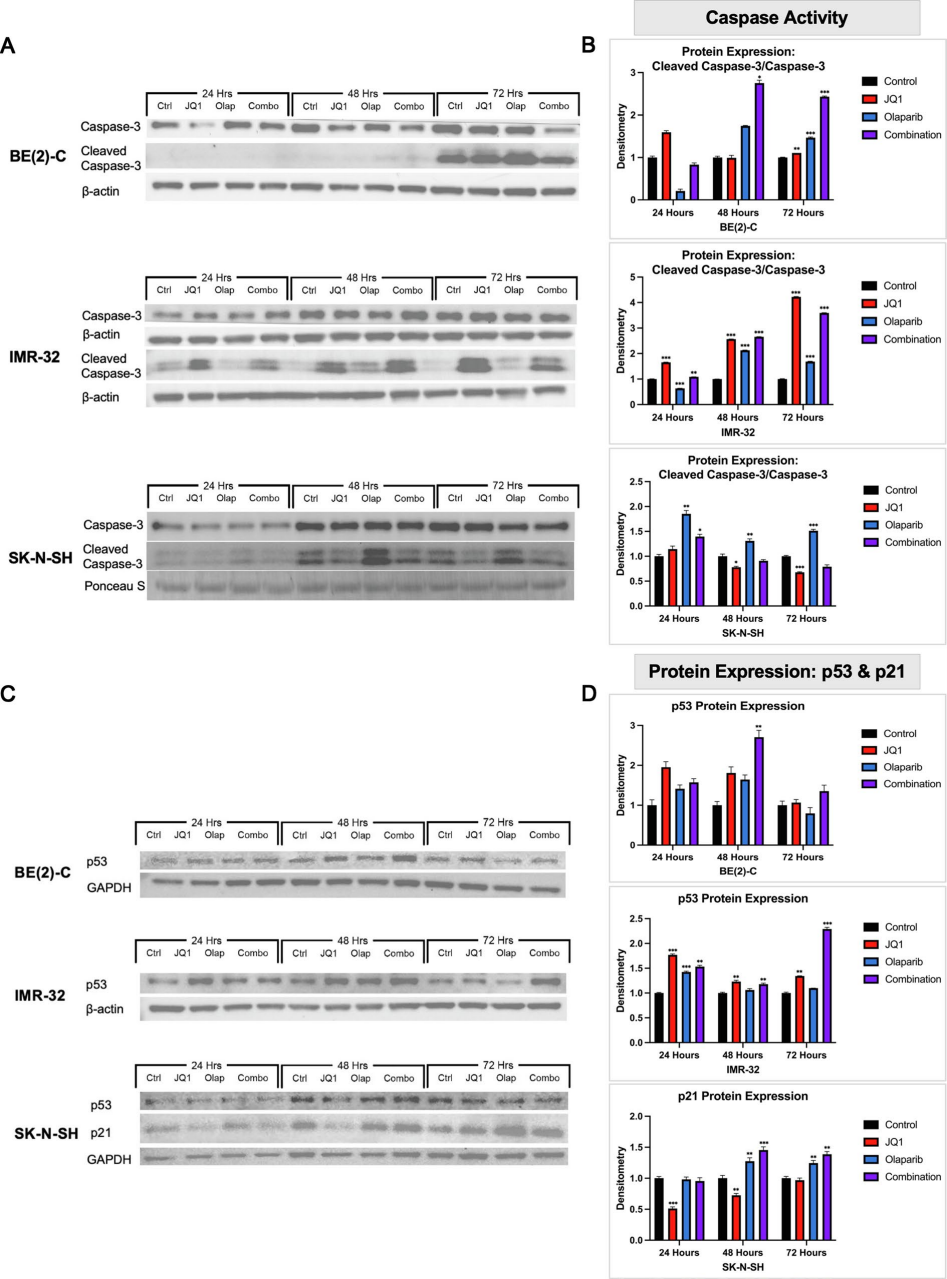
Combination BET and PARP inhibition caused apoptosis in *MYCN*-amplifying NB cells that was associated with accumulation of p53 protein. **A** *MYCN*-amplified BE(2)-C and IMR-32 cells demonstrated increased expression of cleaved caspase-3 protein on Western blotting relative to inactive full-length caspase-3 protein expression beginning 48 and 24 h after combination therapy, respectively, relative to control. This sustained increase was not seen in the non-*MYCN*-amplified SK-N-SH cell line. **B** Densitometry analysis of caspase-3 and cleaved caspase-3 protein expression was performed, analyzed as a ratio of each protein band density relative to the density of each housekeeping control band, and then presented as a ratio of cleaved caspase-3 protein expression to full-length caspase-3 protein expression to represent caspase activity. *MYCN*-amplified cells demonstrated a sustained increase in caspase activity with combination therapy relative to control. This effect was not seen in the SK-N-SH cell line. **C** *MYCN*-amplified BE(2)-C (p53 mutated) and IMR-32 (p53 wild type) cells demonstrated accumulation of p53 protein after combination treatment relative to control on Western blotting. Non-*MYCN*-amplified SK-N-SH cells demonstrated accumulation of p21 protein with combination treatment relative to control. **D** Densitometry analysis of p53 protein expression in the BE(2)-C and IMR-32 cell lines, as well as p21 protein expression in the SK-N-SH cell line are shown. Combination treatment induced an increase in p53 expression in the BE(2)-C and IMR-32 cell lines, as well as an increase in p21 expression in the SK-N-SH cell line. (Mean ± SEM; * = *p* ≤ .05 vs. control, ** = *p* ≤ .01 vs. control, *** = *p* ≤ .001 vs. control)

In order to elucidate the mechanism of inhibition and decreased cell proliferation seen with combination therapy, immunoblotting was performed to assess for markers associated with apoptosis (Fig. 5A). The *MYCN*-amplifying BE(2)-C and IMR-32 cell lines demonstrated increased caspase activity, expressed as a ratio of cleaved caspase-3 to full-length caspase-3 protein, with combination treatment relative to control or single drug treatment beginning 48 and 24 h after treatment, respectively (Fig. 5B). Both cell lines also demonstrated an increase in expression of p53 protein relative to control or single drug treatment (Fig. 5C, D). Interestingly, this increase in p53 and caspase activity was not seen in the non-*MYCN*-amplifying SK-N-SH cell line in response to treatment (Fig. 5A–C).

Given p53’s known role in inducing apoptosis in DNA damaged cells, the data suggest that combination treatment with JQ1 and Olaparib may cause an accumulation of p53 that contributes to apoptosis in *MYCN*-amplifying NB cell lines. However, attributing causality between p53 and induction of apoptosis would be problematic given that BE(2)-C NB cells are known to have a p53 mutation. Therefore, this accumulation of p53 may instead be a surrogate of another process, such as accumulation of DNA damage. These findings prompted the inclusion of the IMR-32 cell line for analysis in this study given its p53 wild type status.

### JQ1 and Olaparib treatment inhibited NB proliferation in the non-*MYCN*-amplified cell lines SK-N-SH and SH-SY5Y via G0/G1 cell cycle arrest and cellular senescence

Given that the SK-N-SH cell line did not demonstrate a sustained increase in caspase activity or an accumulation of p53 protein with combination treatment (Fig. 5A–C), a different mechanism of action may contribute to combination therapy’s anti-cancer activity in non-*MYCN*-amplifying cell lines. After immunoblotting demonstrated SK-N-SH cells’ increase in protein expression of p21 (Fig. 5C), a cyclin-dependent kinase inhibitor that indicates the transcriptional activity of p53 [27], with combination treatment relative to control, we hypothesized that checkpoint-induced cell cycle arrest and cellular senescence may explain the decreased cellular proliferation seen in non-*MYCN*-amplifying cell lines after combination therapy.

**Fig. 6:**
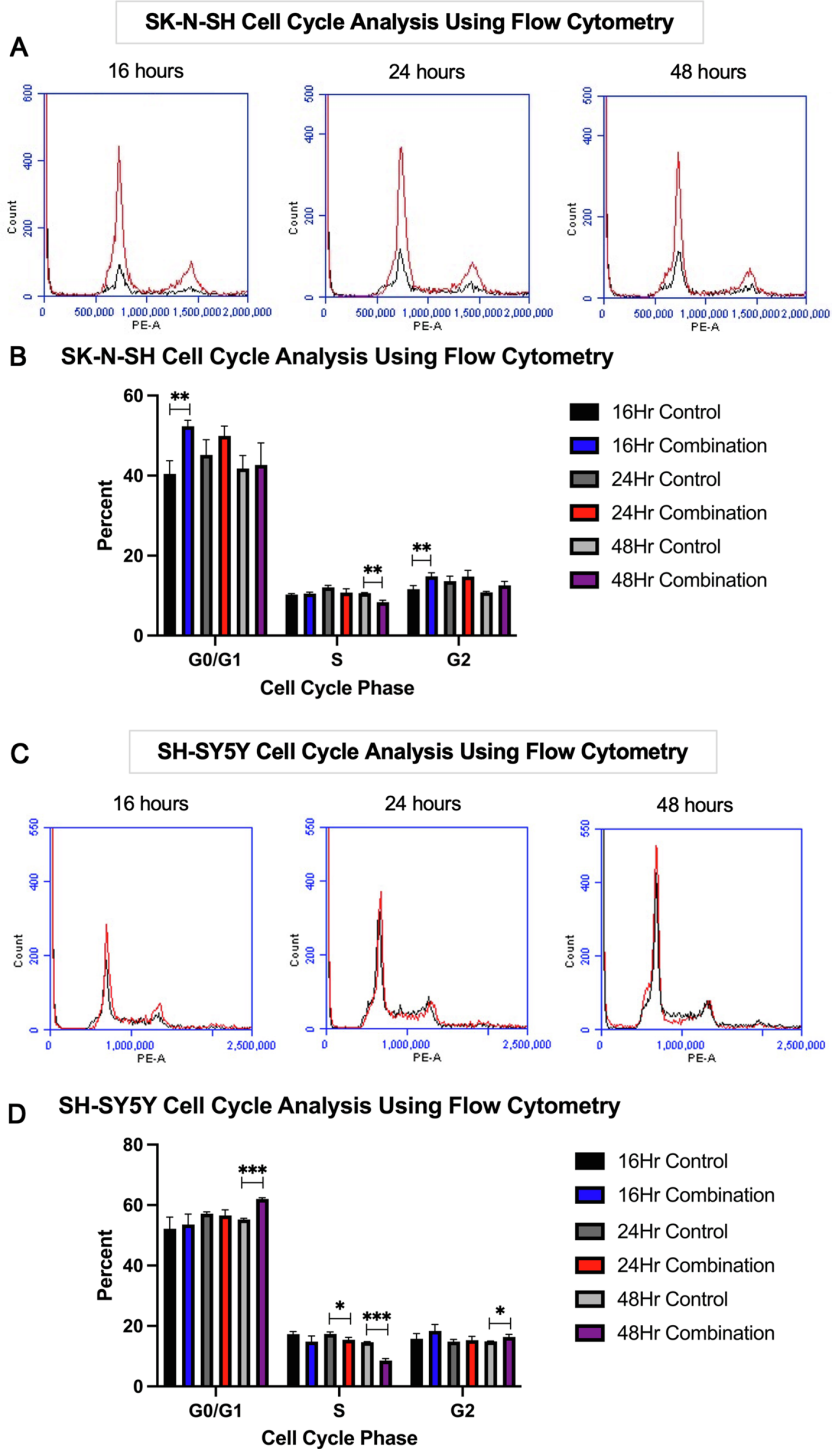
Combination treatment induced cell cycle (G0/G1) arrest in the non-*MYCN*-amplified SK-N-SH and SH-SY5Y cell lines. **A** Cell cycle analysis was performed on SK-N-SH cells using flow cytometry to compare the effects of combination treatment (red line) versus control (black line) at 16, 24, and 48 h after treatment. **B** Combination treatment resulted in an 11.93% ± 2.09% increase in G0/G1 cell cycle arrest at 16 h relative to control (*p* = .01). **C** Cell cycle analysis was performed on SH-SY5Y cells using flow cytometry to compare the effects of combination treatment (red line) versus control (black line) at 16, 24, and 48 h after treatment. **D** Combination treatment resulted in a 6.78% ± 0.30% increase in G0/G1 cell cycle arrest at 48 h relative to control (*p* < .001).

Cell cycle analysis was performed on SK-N-SH cells using flow cytometry (Fig. 6A). After combination treatment, SK-N-SH cells demonstrated an 11.93% ± 2.09% increase in G0/G1 cell cycle arrest at 16 h relative to control (*p* = 0.01, Fig. 6B).

This proposed mechanism of cell cycle arrest was subsequently also tested in the non-*MYCN*-amplifying SH-SY5Y cell line using flow cytometry (Fig. 6C). After combination treatment, SH-SY5Y cells demonstrated a 6.78% ± 0.30% increase in G0/G1 cell cycle arrest at 48 h relative to control (*p* < 0.001, Fig. 6D).

### JQ1 and Olaparib synergistically increased DNA damage in *MYCN*-amplifying NB cells

**Fig. 7:**
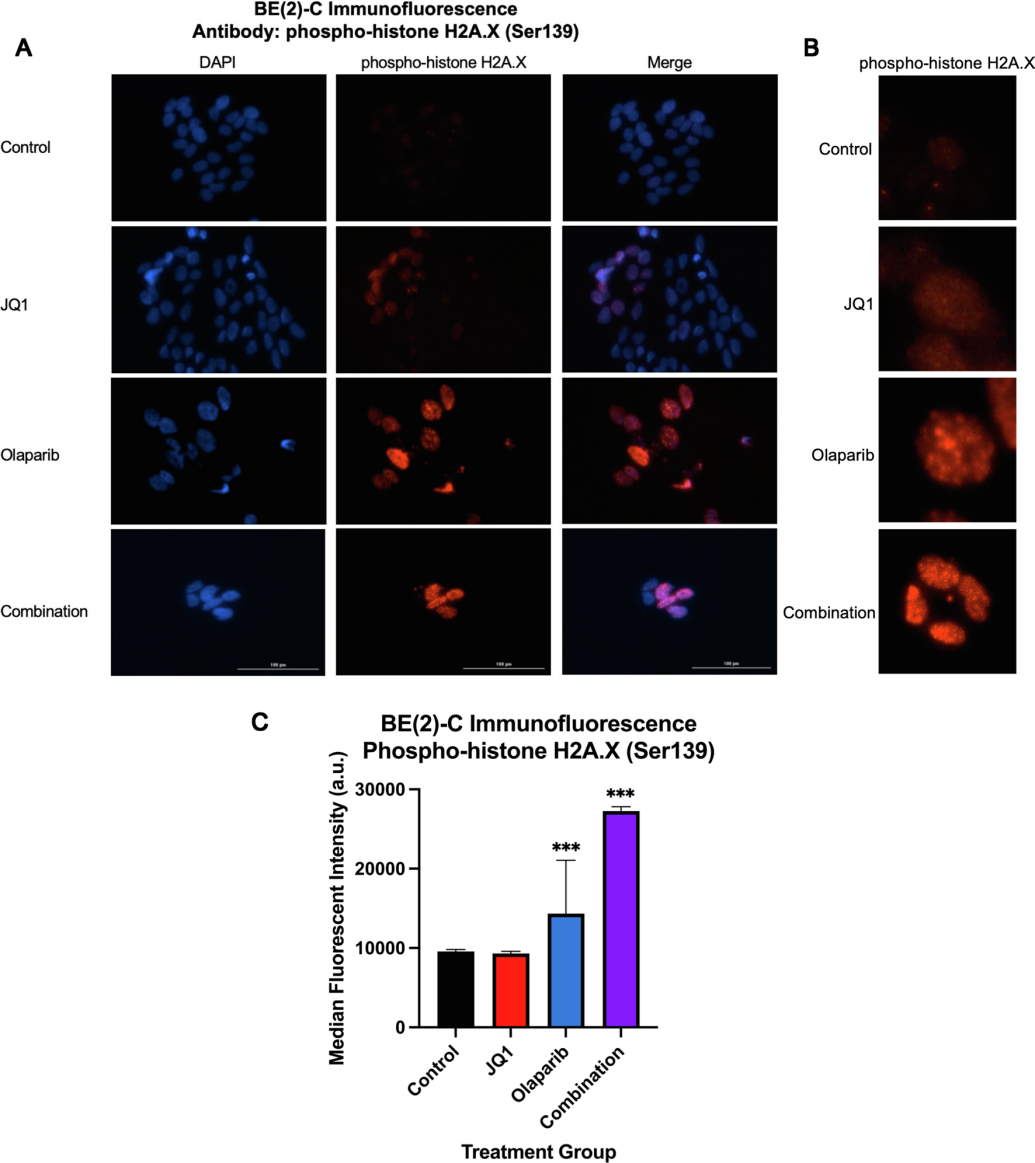
JQ1 and Olaparib functioned synergistically to enhance DNA damage in *MYCN*-amplifying NB cells. **A** Immunofluorescence was performed using antibody against phospho-histone H2A.X (Ser139), a known marker of DNA damage, to evaluate for treatment-induced nuclear DNA damage on BE(2)-C cells separated into four treatment groups: control, JQ1 (1000 nM), Olaparib (3000 nM), or combination treatment. **B** Representative, enlarged images of a single nucleus or a single cluster of nuclei stained with phospho-histone H2A.X are depicted for each treatment group. **C** Immunofluorescence was measured and quantified relative to control. The median (IQR) fluorescence intensity of phospho-histone H2A.X for the control, JQ1, Olaparib, and combination treatment groups were 9551.0 (9154.0–9813.5), 9316.0 (9015.3–9549.0), 14,325.5 (10,525.8–19,253.0), and 27,270.0 (20,287–27,544.5) a.u., respectively. Statistical significance was determined between each treatment group relative to control using Mann–Whitney U tests (* = *p* ≤ .05 vs. control, ** = *p* ≤ .01 vs. control, *** = *p* ≤ .001 vs. control)

To further assess the hypothesis that combination treatment may be associated with apoptosis in the setting of DNA damage in *MYCN*-amplifying cell lines, immunofluorescence was performed on BE(2)-C cells using the phospho-histone H2A.X (Ser139) antibody to evaluate whether JQ1 enhances DNA damage induced by Olaparib treatment. The phosphorylated Ser139 variant of histone H2A.X is a known marker of DNA damage and signifies cellular response to DNA strand breaks [13, 22, 28]. Consistent with previous studies of other cancers, Olaparib alone was sufficient to induce DNA damage; however, DNA damage was relatively enhanced by combination treatment (Fig. 7A–C). The median (IQR) fluorescence intensity of phospho-histone H2A.X for the control, JQ1, Olaparib, and combination treatment groups were 9551.0 (9154.0–9813.5), 9316.0 (9015.3–9549.0), 14,325.5 (10,525.8–19,253.0), and 27,270.0 (20,287–27,544.5) a.u., respectively (Fig. 7C). There was a statistically significant difference in mean and median fluorescent intensity across all four treatment groups (*p* < 0.0001). There was also a statistically significant difference in mean and median fluorescence between control and Olaparib treatment groups (*p* < 0.0001), as well as control and combination treatment groups (*p* < 0.0001).

### DNA damage after combination BET and PARP inhibition was due to downregulated expression of DNA repair proteins required for NHEJ and HR double strand DNA break repair pathways

**Fig. 8:**
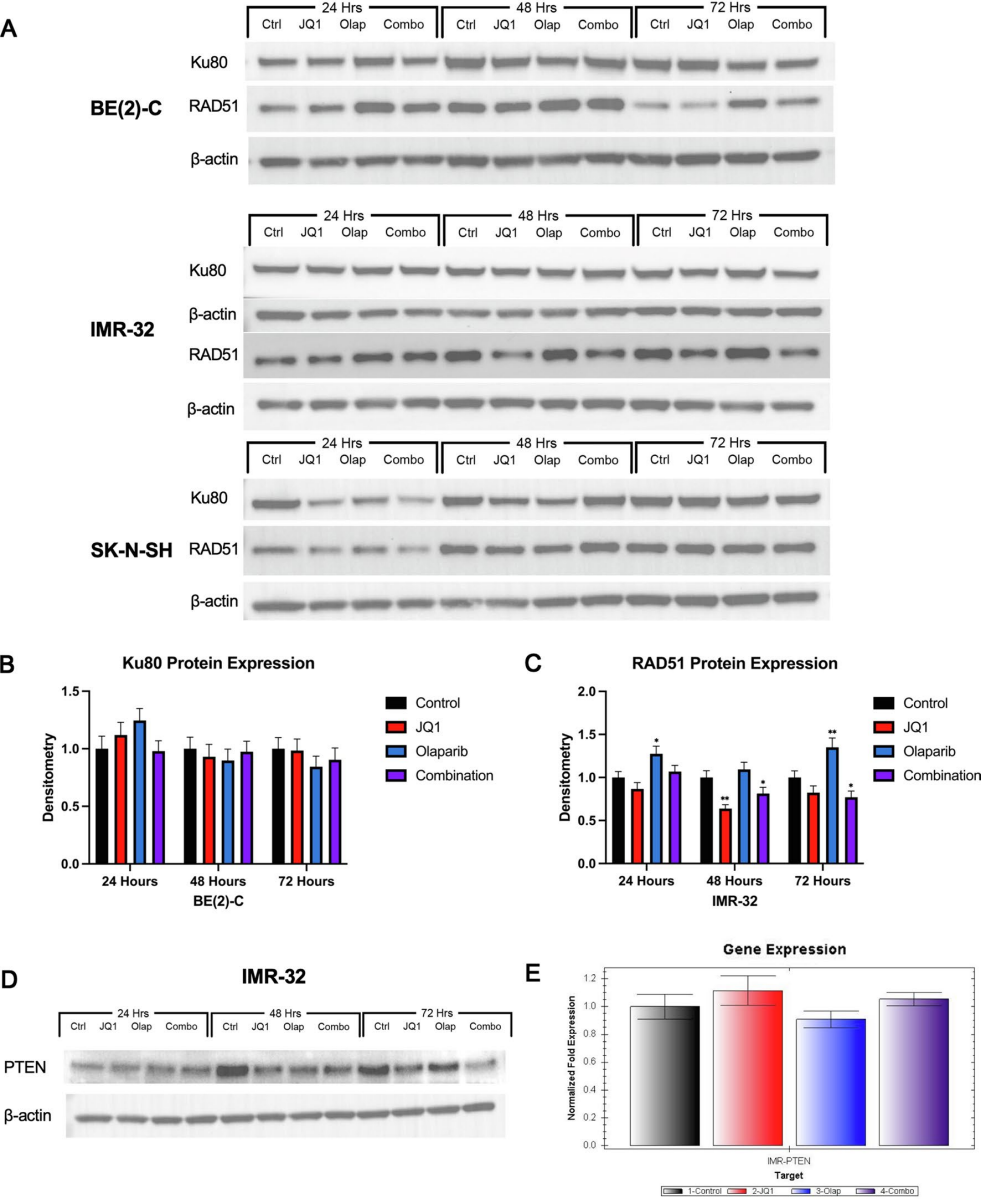
DNA damage after combination BET and PARP inhibition was due to downregulated expression of DNA repair proteins required for NHEJ and HR double strand DNA break repair pathways. **A** Combination BET and PARP inhibition of BE(2)-C cells was associated with a sustained decrease in the expression of Ku80, a DNA damage repair protein required for NHEJ, relative to control on Western blotting. Combination treatment of IMR-32 cells was associated with a sustained decrease in the expression of RAD51, a DNA damage repair protein required for HR, relative to control. Combination treatment of SK-N-SH cells resulted in a decrease in expression of both Ku80 and RAD51 relative to control at 24 h that was not sustained at 48 or 72 h. **B** Densitometry analysis of Ku80 protein expression was performed and presented as a ratio of Ku80 protein band density relative to the density of each β-actin control band. BE(2)-C cells demonstrated a sustained 2.08%, 2.58%, and 9.62% decrease in Ku80 protein expression relative to control at 24, 48, and 72 h after combination treatment, respectively. (**C**) Densitometry analysis of RAD51 protein expression was performed and presented as a ratio of RAD51 protein band density relative to the density of each β-actin control band. IMR-32 cells treated with JQ1 alone demonstrated a 13.3%, 36.1%, and 17.62% decrease in RAD51 protein expression at 24, 48, and 72 h, respectively, relative to control (**A** and **C**). Combination treatment resulted in a 23.0% decrease in RAD51 protein expression at 72 h relative to control. **D** PTEN is known to induce RAD51 expression. The combination treatment-induced decrease in RAD51 protein expression seen in IMR-32 cells was associated with a 32.5% and 68.7% decrease in PTEN protein expression at 48 and 72 h, respectively, relative to control. **E** The IMR-32 cell line demonstrated no significant difference in *PTEN* gene transcription after drug treatment, suggesting that the decrease in PTEN protein expression is limited to the functional level

JQ1 inhibits and regulates BRD4, a member of the BET family of bromodomains and a transcriptional and epigenetic regulator. BRD4 serves as a chromatin platform responsible for gene expression and recruitment of components of DNA repair to sites of DNA-double strand breaks to facilitate DNA damage repair [9]. We therefore hypothesized that JQ1 induces a DNA repair-deficient phenotype that increases susceptibility to Olaparib-induced DNA damage. To further evaluate the mechanism of action of enhanced DNA damage seen with combination treatment, immunoblotting was performed to assess for protein expression of the DNA damage repair proteins Ku80 and RAD51 (Fig. 8A).

It is thought that cells that incur DNA damage marked by 5’-3’ resection of DNA ends will utilize HR mechanisms and prevent NHEJ [29]. RAD51 is a highly conserved protein that facilitates and serves as a marker of DNA repair via HR [30, 31]. Alternatively, binding of the Ku70/Ku80 heterodimer to double strand break ends protects DNA ends against exonucleases, promoting NHEJ and inhibiting HR [29]. HR is generally considered to be a conservative double strand break repair pathway, whereas NHEJ has poor fidelity and is often associated with nucleotide deletions or insertions at repair junctions [32].

With combination treatment, BE(2)-C cells demonstrated a sustained 2.08%, 2.58%, and 9.62% decrease in Ku80 protein expression at 24, 48, and 72 h, respectively, relative to control (Fig. 8B). IMR-32 cells treated with JQ1 alone demonstrated a 13.3%, 36.1%, and 17.62% decrease in RAD51 protein expression at 24, 48, and 72 h, respectively, relative to control (Fig. 8C). Combination treatment resulted in a decrease in RAD51 protein expression of 23.0% at 72 h versus 17.62% with JQ1 treatment alone (Fig. 8C). Conversely, SK-N-SH cells demonstrated only a transient decrease in both Ku80 and RAD51 protein expression at 24 h with combination treatment (Fig. 8A), further suggesting that the mechanism of inhibited tumorigenesis may differ based on *MYCN* amplification status.

Given phosphatase and tensin homolog (PTEN)’s known role in regulation of DNA repair via induction of RAD51 expression [33], we evaluated whether the sustained decrease in RAD51 expression in IMR-32 cells treated with JQ1 and Olaparib was associated with a decrease in PTEN expression. The decrease in RAD51 protein expression seen with combination treatment of IMR-32 cells at 48 and 72 h relative to control was associated with a 32.5% and 68.7% decrease in PTEN protein expression at 48 and 72 h, respectively, relative to control (Fig. 8D).

To determine whether this decrease in PTEN was limited to the functional/protein level or due to changes in gene transcription, RT-qPCR was performed to evaluate for changes in gene expression of *PTEN* after drug treatment. The IMR-32 cell line demonstrated no significant difference in *PTEN* gene expression between treatment groups (Fig. 8E), suggesting that the decrease in PTEN protein expression associated with decreased RAD51 protein expression is occurring at the functional level.

### Combination treatment with JQ1 and Olaparib functioned synergistically against neuroblastoma tumorigenesis in both *MYCN*-amplifying and non-*MYCN*-amplifying cell lines when assessed quantitatively using the median-effect principle of Chou & Talalay

The proposed mechanism of action of combination therapy against neuroblastoma tumorigenesis suggests functional synergy. Olaparib converts single-strand DNA breaks to double-strand DNA breaks, increasing cells’ dependence on NHEJ and HR double-strand break DNA repair pathways. In *MYCN*-amplifying cells, specifically, JQ1’s downregulation of *MYCN* and DNA damage repair proteins Ku80 and RAD51 decreases the efficacy of the double-strand break DNA repair pathways that Olaparib-treated cells are now dependent upon, contributing to an accumulation of DNA damage and ultimately apoptosis. Meanwhile, treatment with Olaparib decreases the IC_50_ of JQ1 regardless of *MYCN*-amplification status.

However, the absence of a standardized definition of therapeutic synergy has historically allowed for additive effects of combination therapy to be mistaken for synergy [34]. We therefore sought to quantitatively assess whether JQ1 and Olaparib combination therapy demonstrates therapeutic synergy against the *MYCN*-amplifying BE(2)-C and non-*MYCN* amplifying SK-N-SH cell lines based on the median-effect principle of Chou and Talalay using Compusyn software (ComboSyn, Inc., Paramus, NJ, USA) [25]. The results of the previously described experiments were input into the Compusyn software. For anti-cancer agents, given that the goal of therapy is tumor eradication, combination index (CI) values indicating synergy at higher doses and high treatment effect (i.e., Fa) are considered more relevant to therapy than CI values at lower doses and low treatment effect [25]. Fa ranges from 0 to 1 and refers to the fraction of cells affected (e.g., Fa = 0.5 signifies 50% inhibition of cell viability) [25]. Therefore, experiments with at least two data points and the widest range of treatment doses were selected for analysis. As described by Chou & Talalay, a CI less than 1 was considered indicative of synergy (additive effect: CI = 1, antagonistic effect: CI > 1) [25, 35].

Both the *MYCN*-amplifying BE(2)-C and non-*MYCN* amplifying SK-N-SH cell lines demonstrated therapeutic synergy at increasing Fa values as demonstrated by CIs less than 1. For instance, combination treatment of BE(2)-C cells with JQ1 and Olaparib at their approximate IC_50_ dosages led to an Fa = 0.509 and CI = 0.33, signifying therapeutic synergy. Furthermore, at these treatment doses and Fa = 0.509, dose-reduction index (DRI) data demonstrated that combination treatment required 3.9-fold less JQ1 plus 14.0-fold less Olaparib to achieve the same 50% inhibition in a 1:1 combination in comparison to the doses required of each drug alone. However, combination treatment at lower doses yielding lower Fa did not consistently demonstrate a CI less than 1. Similarly, combination treatment of SK-N-SH cells with lower doses of JQ1 and Olaparib yielding lower Fa (0.3–0.5) was associated with CIs of 0.95–1. However, higher doses yielding a higher Fa of 0.7 were associated with a substantial decrease in CI to 0.45. Furthermore, this was associated with a DRI of 2.2-fold less JQ1 plus 374.7-fold less Olaparib to achieve the same 70% inhibition in a 1:1 combination in comparison to the doses required of each drug alone. This data suggest that combination treatment of both *MYCN*-amplifying and non-*MYCN*-amplifying cells with doses of JQ1 and Olaparib sufficient to yield higher treatment effect are associated with true synergy and substantial dose-reduction indices compared to single drug treatment.

## Discussion

Patients with high-risk NB undergo multimodality treatment consisting of multi-agent chemotherapy induction, surgical resection, consolidation chemotherapy, autologous stem cell rescue, and radiation therapy followed by treatment with anti-ganglioside-2 (anti-GD2) immunotherapy, cytokines, and cis-retinoic acid [36]. Despite this aggressive and often highly morbid treatment, high-risk NB remains difficult to treat and continues to account for approximately 15% of pediatric cancer deaths with a five-year survival rate of less than 40% [1]. Furthermore, many patients, including those who initially demonstrated tumor regression with current standard therapies, later demonstrate tumor recurrence that is often marked by resistance to established chemotherapy agents and radiation [2]. Therefore, there is a need to develop and utilize new agents and combinatorial therapy strategies for the treatment of NB.

In preclinical models of NB, BET inhibition has been shown to downregulate *MYCN* expression, induce differentiation toward a neural fate, prevent proliferation, and induce cell cycle arrest and apoptosis [5, 6, 10, 11]. PARP inhibition has been demonstrated to inhibit checkpoint inhibition in *MYCN*-dependent NB cells, causing progression through the cell cycle despite DNA damage and ultimately mitotic catastrophe and apoptosis [16, 37]. Widespread clinical utilization of each of these treatment strategies individually, however, has been limited. The clinical use of BET inhibition for various cancer types has been limited by dose-limiting toxicities [7]. The use of PARP inhibition in NB has also been relatively limited. Existing studies of PARP inhibition in NB have largely established its efficacy in specific tumor types, including those that amplify or overexpress *MYCN*, as well as those with 11q deletion and/or low ataxia-telangiectasia mutated (ATM) expression [16, 37–42]. Given the significant heterogeneity in NB tumors, it is possible that this has led to doubt over its widespread efficacy in other NB tumor types. Our results demonstrate that combination treatment strategies can both overcome the weaknesses of either drug class as a monotherapy and work synergistically to provide greater anti-cancer efficacy than either drug alone.

A combination of BET and PARP inhibition has demonstrated efficacy in inhibiting tumorigenesis in preclinical models of numerous malignancies, including pancreatic, breast, ovarian, prostate, and lung cancers [13, 15, 17, 22]. Given previous reports of NB’s susceptibility to BET and PARP inhibitors in preclinical models testing each drug class individually [5, 6, 10, 11, 16], we hypothesized that JQ1, a BET inhibitor, would promote a DNA repair-deficient phenotype that would function synergistically with Olaparib, a PARP inhibitor, to inhibit NB tumorigenesis. Our results demonstrate that combination BET and PARP inhibition functions synergistically against NB tumorigenesis in vitro with greater efficacy than either drug alone. Furthermore, these synergistic effects are seen regardless of *MYCN* amplification status, although the mechanism may vary.

In *MYCN*-amplified NB cell lines, combination treatment was associated with downregulation of *MYCN* gene expression and N-myc protein expression, downregulation of DNA repair proteins, accumulation of p53 and DNA damage, and induction of apoptosis. This proposed mechanism of downregulation of the DNA damage response contributing to synergistic cytotoxicity is consistent with preclinical studies of other cancer types, including pancreatic, ovarian, breast, prostate, and lung cancers [13, 15, 17, 22].

Previous preclinical studies of BET and PARP inhibitors have largely focused on cancer cell lines with *MYC* amplification. Fiorentino et al. concluded that combination BET and PARP inhibition demonstrated preferential or selective inhibition on small cell lung cancer cells with an active *MYC* signaling pathway [17]. However, we found that non-*MYC*-amplified NB cells remain susceptible to combination treatment, albeit seemingly through a different mechanism of action. Lee et al. demonstrated that JQ1 alone was sufficient to halt proliferation in the SK-N-SH cell line [5]. Wyce et al. also found that BET inhibition induced a concentration-dependent G1 arrest in the non-*MYCN*-amplifying SK-N-SH and SK-N-AS cell lines [43]. Given that SK-N-SH demonstrated an increase in p21 protein expression with combined BET and PARP inhibition treatment in this study, we hypothesized that cell cycle arrest may be contributing to the inhibition in cell proliferation seen in non-*MYCN*-amplifying cell lines. Our results supported this hypothesis, with an 11.93% increase in G0/G1 cell cycle arrest at 16 h and a 6.78% increase in G0/G1 cell cycle arrest at 48 h in the SK-N-SH and SH-SY5Y cell lines, respectively, after combination treatment relative to control. This data suggest that combination treatment may affect cell cycle progression and alter checkpoint inhibition.

The results of this study have substantial potential translational relevance. Several PARP inhibitors, including Olaparib, Rucaparib, Talazoparib, and Niraparib, have already been approved by the FDA for the treatment of other cancers [41, 44]. Numerous BET inhibitors, including oral agents, are currently in early phase clinical trials for the treatment of both hematological and solid tumor malignancies [7]. Therefore, if future clinical trials demonstrate anti-cancer effectiveness of combination BET and PARP inhibition treatment against neuroblastoma, this treatment strategy could be more rapidly implemented relative to other novel, untested, or unapproved agents.

The BET and PARP inhibition combination treatment strategy may also aid in overcoming other challenges in cancer treatment, such as treatment resistance. Our results demonstrated that BET inhibition sensitized neuroblastoma cells to PARP inhibition in vitro. These results are consistent with studies of other cancer types. PARP inhibition is a known clinical strategy against *BRCA*-mutated ovarian cancers. However, Karakashev et al. demonstrated that combination treatment with BET inhibition suppressed TOPBP1 and WEE1 expression, resulting in accumulation of DNA damage, checkpoint defects, and apoptosis in preclinical models of *BRCA*-wild type ovarian cancer [15].

BET inhibition may also work synergistically with other agents and treatment modalities. BET inhibition downregulates RAD51, whose overexpression has been associated with chemoresistance in multiple cancer types, including neuroblastoma [45–47]. In addition, Li et al., in a preclinical model of prostate cancer, found that BET inhibition’s downregulation of RAD51 and DNA damage repair both enhanced radiation effect and aided in overcoming radioresistance [8]. Given that chemotherapy and radiation are vital modalities in the treatment of high-risk NB, these findings could have potential important clinical significance in NB treatment outcomes, both during initial treatment and in cases of relapse marked by chemo- and radioresistance.

This study is limited by its preclinical design. Further work is needed to test the safety and effectiveness of combination BET and PARP inhibition clinically. Another limitation is that BET inhibition likely affects the expression of other genes and proteins not tested in the current study that may affect or correlate with the observed synergistic effects of BET and PARP inhibition. Future directions include evaluating the effects of BET and PARP inhibition on other therapeutic agents and treatment modalities, such as radiation therapy, used in the management of NB.

In conclusion, the combination of BET and PARP inhibition synergistically inhibited NB tumorigenesis in vitro. These results were seen regardless of *MYCN* amplification status; however, the mechanism of action may vary. In *MYCN*-amplified cell lines, combination treatment was associated with downregulation of *MYCN* transcription, defects in DNA repair, accumulation of DNA damage, and apoptosis. In non-*MYCN*-amplified cell lines, combination treatment induced cell cycle arrest. A combination of BET and PARP inhibition is a potentially novel, efficacious treatment strategy in the management of NB.

## Supplementary Information

**Additional file 1: Figure S1. **Staggering combination treatment with Olaparib 24 h after treatment with JQ1 has variable response on the IC_50_ of JQ1 by cell line. When staggering treatment of Olaparib 24 h after treatment of JQ1, the IC_50_ of JQ1 increased 6.4-fold in the BE(2)-C cell line (Fig. S1A) and decreased 1.7-fold and 51.3-fold in the IMR-32 (Fig. S1B) and SK-N-SH (Fig. S1C) cell lines, respectively.

## Data Availability

The datasets generated during and/or analyzed during the current study are available from the corresponding author upon reasonable request.

## Abbreviations

Anti-GD2: Anti-ganglioside-2
ATM: Ataxia-telangiectasia mutated
a.u.: Arbitrary units
BET: Bromo- and extraterminal (domain)
BRD: Bromodomain *or* bromodomain-containing protein (when referring to BRD2, BRD3, BRD4)
BRDT: Bromodomain testis-specific protein
CCK-8: Cell Counting Kit-8
CI: Combination index
DAPI: 4’,6-Diamidino-2-phenylindole
DRI: Dose-reduction index
Fa: Fraction of cells affected
FBS: Fetal bovine serum
FDA: Food and Drug Administration
HR: Homologous recombination
IQR: Interquartile range
NB: Neuroblastoma
NF-M: Neurofilament-medium
NHEJ: Non-homologous end-joining
NSE: Neuron-specific enolase
PARP: Poly (ADP-ribose) polymerase
PBS: Phosphate-buffered saline
PTEN: Phosphatase and tensin homolog
RPMI: Rockwell Park Memorial Institute
RT-qPCR: Reverse transcription-quantitative polymerase chain reaction
SD: Standard deviation
SEM: Standard error of the mean
