# Supplementary figures and images for: Combination bromo- and extraterminal domain and poly (ADP-ribose) polymerase inhibition synergistically enhances DNA damage and inhibits neuroblastoma tumorigenesis
Source: Discov Oncol. 2022 Oct 13;13:103. doi: 10.1007/s12672-022-00563-5 (PMC9562984; doi:10.1007/s12672-022-00563-5)

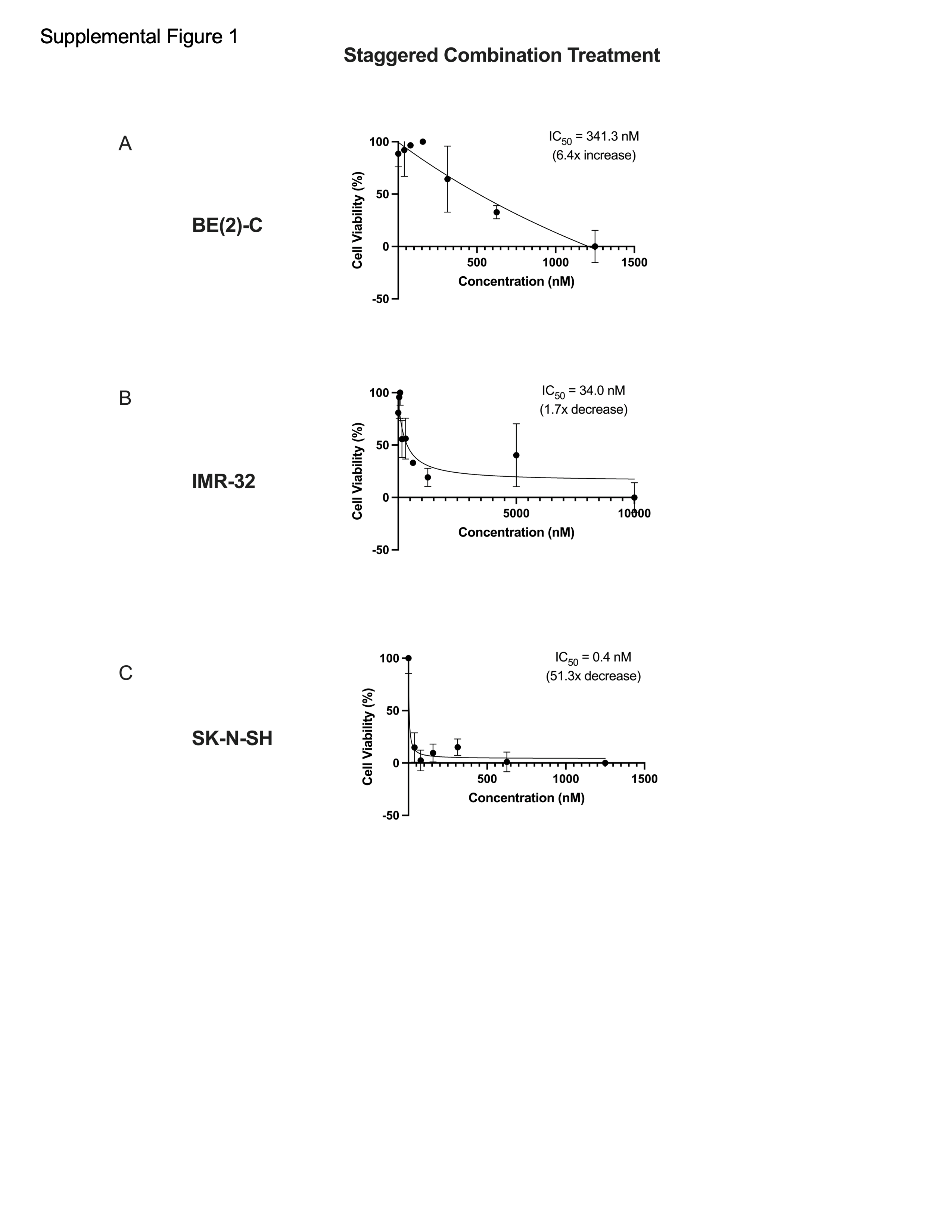

Supplement: Supplementary file 1 — Additional file 1: Figure S1. Staggering combination treatment with Olaparib 24 h after treatment with JQ1 has variable response on the IC50 of JQ1 by cell line. When staggering treatment of Olaparib 24 h after treatment of JQ1, the IC50 of JQ1 increased 6.4-fold in the BE(2)-C cell line (Fig. S1A) and decreased 1.7-fold and 51.3-fold in the IMR-32 (Fig. S1B) and SK-N-SH (Fig. S1C) cell lines, respectively. [file 12672_2022_563_MOESM1_ESM.tiff]
